# Mesenchymal stem cell-derived exosomes as a plausible immunomodulatory therapeutic tool for inflammatory diseases

**DOI:** 10.3389/fcell.2025.1563427

**Published:** 2025-03-10

**Authors:** Muhammad Zubair, Fatma A. Abouelnazar, Muhammad Asad Iqbal, Jingyun Pan, Xuwen Zheng, Tao Chen, Wenming Shen, Jinnan Yin, Yongmin Yan, Pengjun Liu, Fei Mao, Ying Chu

**Affiliations:** ^1^ Department of Laboratory Medicine, Wujin Hospital Affiliated with Jiangsu University, Changzhou, China; ^2^ Wujin Institute of Molecular Diagnostics and Precision Cancer Medicine of Jiangsu University, Wujin Hospital Affiliated with Jiangsu University, Changzhou, China; ^3^ Faculty of Applied Health Sciences Technology, Pharos University, Alexandria, Egypt; ^4^ School of Medicine, Jiangsu University, Zhenjiang, Jiangsu, China; ^5^ Department of Traditional Chinese Medicine, Wujin Hospital Affiliated with Jiangsu University, Changzhou, China; ^6^ Department of Emergency, Wujin Hospital Affiliated With Jiangsu University, Changzhou, China; ^7^ Department of Gastroenterology, Wujin Hospital Affiliated With Jiangsu University, Changzhou, China; ^8^ Key Laboratory of Medical Science and Laboratory Medicine of Jiangsu Province, Department of Laboratory Medicine, School of Medicine, Jiangsu University, Zhenjiang, Jiangsu, China; ^9^ Wujin Clinical College, Xuzhou Medical University, Changzhou, China; ^10^ Jiangsu Key Laboratory of New Drug Research and Clinical Pharmacy, Xuzhou Medical University, Xuzhou, China

**Keywords:** exosomes, immunomodulation, inflammatory diseases, mesenchymal stem cells, therapeutics

## Abstract

Mesenchymal stem cell-derived extracellular vesicles (MSC-EVs), especially, exosomes are considered to have diverse therapeutic effects for various significant diseases. MSC-derived exosomes (MSCex) offer substantial advantages over MSCs due to their long-term preservation, stability, absence of nuclei and fewer adverse effects such as infusion toxicity, thereby paving the way towards regenerative medicine and cell-free therapeutics. These exosomes harbor several cellular contents such as DNA, RNA, lipids, metabolites, and proteins, facilitating drug delivery and intercellular communication. MSCex have the ability to immunomodulate and trigger the anti-inflammatory process hence, playing a key role in alleviating inflammation and enhancing tissue regeneration. In this review, we addressed the anti-inflammatory effects of MSCex and the underlying immunomodulatory pathways. Moreover, we discussed the recent updates on MSCex in treating specific inflammatory diseases, including arthritis, inflammatory bowel disease, inflammatory eye diseases, and respiratory diseases such as asthma and acute respiratory distress syndrome (ARDS), as well as neurodegenerative and cardiac diseases. Finally, we highlighted the challenges in using MSCex as the successful therapeutic tool and discussed future perspectives.

## 1 Introduction

Mesenchymal stem cells (MSCs) are adult stem cells that have the capability to differentiate into multiple cell types (Pittenger et al., 1999; Oishi et al., 2009; Jiang et al., 2007). These can be obtained from various sources such as bone marrow, umbilical cord, placenta, and adipose tissues. Some unconventional sources are also utilized for the isolation of MSCs, including amniotic fluid, dental pulp, endometrial, tonsils, salivary gland, urine, menstrual blood, peripheral blood, synovial fluid, as well as numerous human tissues such as kidney, liver, and pancreas (Ren et al., 2016; Tavakoli et al., 2020). Regardless of their origins, MSCs retain two crucial inherent traits: the capacity for self-renewal and the capability to differentiate into several cell lineages. MSCs have garnered significant interest as a potential cell-based therapy for human illnesses due to their capacity for differentiation, self-renewal, and immuno-modulation. MSCs express nestin and NANOG genes, which are important indicators for preserving pluripotency and the ability to regenerate (Li et al., 2018). MSCs engage with parenchymal cells and facilitate the restoration and rejuvenation of damaged tissues through direct cell-cell contact and the release of signaling molecules (Volarevic et al., 2017). Damage-associated molecular patterns (DAMPs) and alarmins, which are generated by damaged cells, trigger the activation of MSCs. This activation, in turn, prevents apoptosis of unaffected parenchymal cells promoting survival and multiplication. MSCs inhibit the inflammatory events taking place due to monocytes neutrophils, T lymphocytes, natural killer (NK), and natural killer T (NKT) cells, stimulating the production and development of immunosuppressive T regulatory cells (Tregs), resulting the reduction of inflammation (Harrell et al., 2019).

MSCs generate various EVs which broadly include several forms of vesicles, such as exosomes, micro-vesicles, and apoptotic bodies (Ćulum et al., 2021; Colombo et al., 2014). Exosomes are membranous EVs measuring 30–150 nm in diameter (Johnstone et al., 1987; Baietti et al., 2012). Nevertheless, the term “exosome” specifically refers to vesicles generated within multi-vesicular bodies (MVBs) inside cells (Hade et al., 2021). Initial endosomes are formed through the invagination of the cell membrane, during which bioactive chemicals start to collect within the early sorting endosomes. The late sorting endosomes then develop into MVBs following a second indentation. Ultimately, the MVBs merge with the cell membrane, discharging the encapsulated exosomes externally (Pegtel and Gould, 2019). Exosomes are released by majority of cell types, including immune cells (B cells, T cells, dendritic cells, mast cells), endothelial cells, neuronal cells, embryonic cells, epithelial cells cancer cells, and MSCs (Yu et al., 2014). Exosomes are distinct from micro-vesicles (150–500 nm in diameter), and apoptotic bodies (800–5,000 nm in diameter) (Heldring et al., 2015; Lotfy et al., 2023). These are crucial for intercellular communication as they encapsulate and transmit essential physiologically active chemicals, altering the activity of target cells through several mechanisms (Van Niel et al., 2006). EVs, including exosomes, can encompass many cellular contents such as DNA, RNA, lipids, metabolites, and both cytosolic and cell surface proteins (Deng et al., 2018; Toh et al., 2018; Kalluri and LeBleu, 2020; Qiu et al., 2019). Among these, miRNAs play significant role in inter cellular communications and contribute in the mechanisms involving cell death and cell growth hence paving the way for the cell free therapy of inflammatory diseases (Asgarpour et al., 2020; Giunti et al., 2021). Cytokines are found not only within exosomes but also integrated into the exosomal membrane. Exosomes can transport minor, targeted quantities of cytokines directly to specific cells, providing a more efficient delivery mechanism than the conventional release of cytokines into the intercellular space, where they may be taken up by any cell possessing the precise receptor (Urbanelli et al., 2013; Fitzgerald et al., 2018). Moreover, additional proteins within the exosomal membrane, such as diverse heat-shock and signaling proteins, have demonstrated immunomodulatory effects (Reddy et al., 2018).

MSCex can play vital role in drug delivery system by transporting exogenous chemicals and biomolecules for cell free therapeutics (Phinney and Pittenger, 2017). These exosomes possess numerous potential therapeutic benefits in comparison to manufactured nanoparticles, liposomes, individual compounds, and cells (Heldring et al., 2015; Mendt et al., 2019). This arises from their advantageous traits, including reduced size, diminished complexity, absence of nuclei, enhanced stability, remarkable biocompatibility, simplified production, extended preservation, and ability to carry diverse array of contents including encapsulating proteins, small molecules, or RNAs for bio-molecular delivery (Théry et al., 2002; Tan et al., 2024; Forsberg et al., 2020).

This review cites the recent updates on the role of MSCex in specific inflammatory diseases followed by subsequent mechanisms, challenges and future perspectives. The literature was mined from PubMed and the terminology used for searching the relevant articles were; mesenchymal stem cells exosomes, MSC exosomes and inflammatory diseases, MSC exosomes and inflammatory bowel disease (IBD), MSC exosomes and arthritis, and MSCex in therapeutics.

## 2 Mechanisms of MSCex action in inflammatory diseases

### 2.1 Immunomodulation

**FIGURE 1:**
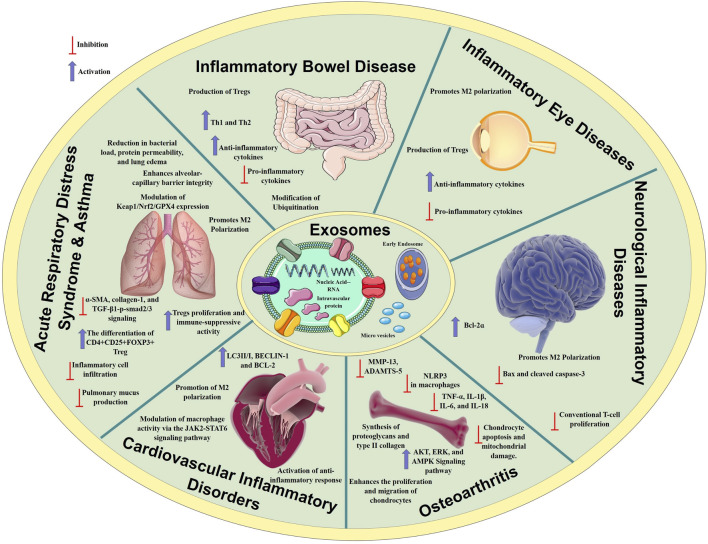
Mechanisms of anti-inflammatory processes regulated by MSCex in various inflammatory diseases. MSCex modulate immune responses by suppressing pro-inflammatory cytokines, promoting anti-inflammatory cytokines release, and modulating immune cell activity. These immuno-modulatory mechanisms contribute to alleviate inflammation in conditions such as osteoarthritis, inflammatory bowel disease, inflammatory eye diseases, neurological and respiratory diseases.

MSCex have been extensively studied for their immunomodulatory effect on both innate and adaptive responses. The role of exosomes encompasses various facets, including immunological response, antigen presentation, cellular motility, and cellular differentiation (Xunian and Kalluri, 2020). Exosomes can initiate signal transmission via receptor-ligand interactions or endocytosis in recipient cells, facilitating the delivery of physiologically active chemicals, cytokines, chemokines, and immuno-regulatory factors to modulate cellular activity (Bazzoni et al., 2020). Figure 1 represents a general illustration of immunomodulatory effects of MSCex in relieving inflammatory diseases.

#### 2.1.1 B Cells

**FIGURE 2:**
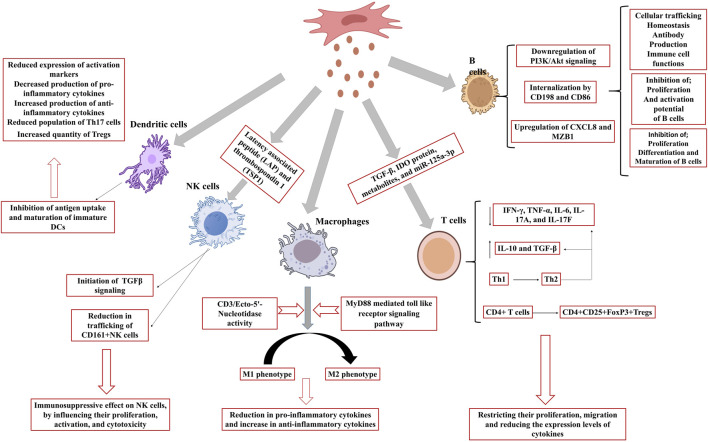
Effect of MSCex on immune cells. MSCex inhibit the proliferation, maturation and activation potential of B cells, activates macrophages polarization, initiates TGFβ signaling in NK cells and inhibits the antigen uptake and maturation of immature DCs to accelerate the anti-inflammatory response.

MSCex impede the proliferation of certain immune cell types, notably B-lymphocytes. Study revealed that variable expression of mRNA demonstrated 39 upregulated and 11 downregulated genes. Among these, CXCL8 and MZB1 had elevated levels and were biologically related genes. This differential expression influences cellular trafficking, development, homeostasis, and immune cell functionality (Khare et al., 2018). MSCex were internalized by activated CD19+/CD86+ B cells, resulting in the inhibition of B cell proliferation, differentiation, antibody production, and maturation of memory B cells (Budoni et al., 2013). Moreover, MSCex demonstrate dose-dependent anti-inflammatory effects by suppressing B cell maturation and promoting Bregs in lymph nodes within a mouse model of collagen-induced arthritis and delayed-type hypersensitivity (Carreras-Planella et al., 2019). Other researchers have also reported a decrease in IgG production, indicating that MSCex may enhance the CD19CIL-10C Breg-like population and suppress the differentiation of plasmablast through the transmission of TGF-β, PEG2, and IL1RA (Cosenza et al., 2017). MSCex prompted B cells to downregulate the PI3K/Akt signaling pathway via miR-155-5p, hence inhibiting B cell proliferation and diminishing the activation potential of B lymphocytes (Adamo et al., 2019) (Figure 2). Another study showed that MSCex suppress the proliferation and differentiation of B cells from 29.4% to 14% and from 59.3% to 45.7% respectively but the suppression effect was relatively lesser to MSCs (Conforti et al., 2014).

#### 2.1.2 T Cells

T cell growth and activation play a significant role in the onset and progression of various diseases, including autoimmune disorders. MSCex adversely affect T cell proliferation and activation resulting in immunosuppressive response (Blazquez et al., 2014; Shen et al., 2021). MSCex diminish immunological responses caused by pro-inflammatory cells by restricting their migration and reducing the expression levels of cytokines produced by these cells, hence alleviating inflammation (Chen et al., 2016; Baharlooi et al., 2022). MSCex have demonstrated the presence of many active molecules, such as TGF-β, IDO protein, metabolites, and miR-125a-3p (Showalter et al., 2019; Romani et al., 2015; Cerwenka and Swain, 1999). A study demonstrated that MSCs and MSCex mitigate the detrimental activity of immunological T cells in patients with multiple sclerosis, principally by diminishing the synthesis of inflammatory cytokines like interferon-γ and interlukin-17 (IL-17). Particularly, MSCex were more efficacious than the MSCs themselves in regulating T cell activity and enhancing anti-inflammatory chemicals such as IL-10 (Fattore et al., 2015). Although, MSCs and their derived exosomes both have immunosuppressive effect but these may act in different fashion. While MSCs diminish T cell proliferation, exosomes augment T cell mortality and facilitate the expansion of regulatory T cells, which assist in suppressing the immunological response (Baharlooi et al., 2021). Interaction of MSCex with other immune cells may improve their immune regulatory action. For example, immune system in graft-versus-host disease mouse model was stimulated with antigen presenting cells followed by the treatment with MSCex. MSCex mitigating the illness symptoms and enhancing the survival by facilitating Treg formation (Fattore et al., 2015), and stimulating the synthesis of anti-inflammatory cytokines such as IL-10 and TGF-β, while diminishing pro-inflammatory cytokines (Zhang B. et al., 2018). In a murine model of allogeneic skin graft, exosome-treated THP-1 cells induced the polarization of activated CD4^+^ T cells into CD4^+^CD25+FoxP3+ regulatory T cells (Tregs) resulting immune suppression and increased survival of mice (Zhang et al., 2014). Another study on Primary Sjogren’s syndrome indicated that exosomes originated from umbilical cord MSCs (UcMSCs) diminished the aberrant proliferation and apoptosis of CD4^+^ T cells. The equilibrium between pro-inflammatory Th17 cells and Tregs was reestablished and decreased the secretion of inflammatory cytokines including IFN-γ, TNF-α, IL-6, IL-17A, and IL-17F (Ma et al., 2023). Also, transgene-free human induced pluripotent stem cells (iPSCs) originated exosomes were discovered to block the differentiation of follicular helper T (Tfh) and Th17 cells, hence preventing the course of Sjögren’s syndrome (Hai et al., 2018). Similarly, exosomes were co-cultured with peripheral blood mononuclear cells and it was concluded that exosomes might facilitate the conversion of Th1 to Th2 helper T cells, markedly diminish the levels of pro-inflammatory molecules IL-1ɑ and TNF-ɑ, and enhance the levels of the anti-inflammatory factor TGF-β (Chen et al., 2016) (Figure 2). MSCex taken from a diseased host may not be as effective as the exosomes originated from the MSCs taken from healthy donor. A study validated the impact of exosomes derived from aplastic anemia patients (AA-Exo) and those from healthy donors (HD-Exo) on immunological T cells. Exosomes from healthy donors shown superior efficacy inhibiting T cell proliferation and activation, in addition to ameliorating a murine model of aplastic anemia (Wang et al., 2023). Certain miRNAs, such as miR-199, miR-128, and miR-486, that participate in immunological responses were also identified (Wang et al., 2023). It was demonstrated that exosomes produced from early passage MSCs inhibit Th1 and Th17 cytokines in splenocyte cultures and ameliorate inflammatory dry eye illness in mice affected with ocular Sjögren’s syndrome. It was observed that levels of TGF-β1, PTX3, and let-7b-5p, were higher in the exosomes originated from early passaged MSCs as compared to those from late-passage MSCs (Kim et al., 2020). This study showed that passages also play a major role in immunomodulatory effect of MSCex. Several studies have demonstrated the immunosuppressive role of MSCex (Blazquez et al., 2014; Shen et al., 2021; Mohammadzadeh et al., 2014; Ji et al., 2019; Du et al., 2018).

#### 2.1.3 Macrophages

Macrophages are innate immune cells that play a crucial role in immune response and tissue repair. MSCex can modulate the development of macrophages into pro-inflammatory M1 or anti-inflammatory M2 phenotypes. M1 macrophages release pro-inflammatory substances, such as TNF-ɑ and IL-1β, while M2 macrophages produce immune regulating factors such as IL-10 and TGF b1 (Qian et al., 2021). Studies have validated various pathways involved in macrophage polarization. These include MyD88 mediated toll like receptor signaling pathway (Zhang et al., 2014) and CD3/Ecto-5′-Nucleotidase activity (Teo et al., 2023). Adipose-derived mesenchymal stem cells (AD-MSCs) influence the immune system by promoting macrophage polarization towards an M2 phenotype, which is linked to anti-inflammatory responses. The induced macrophages (iMΦ) exhibited improved capabilities suppressing activated T cell proliferation and markedly elevated the population of Tregs. Induced macrophages also generated elevated levels of anti-inflammatory cytokines, including IL-10 and TSG-6. Exosomes derived from AD-MSCs also facilitated M2 macrophage polarization (Heo et al., 2019) (Figure 2). Several studies have been documented elaborating the role of MSCex in macrophage polarization (Table 1).

**TABLE 1:** Role of MSCex in macrophage polarization leading to the improved therapeutics.

MSCex	Macrophage polarization mediator	Model	Function	Ref.
Collagen scaffold exosomes (CS/EX)	CD163+M2 polarization mediated by MiRNA	*In vitro/in vivo* mice model	Endometrial trauma/Regeneration, collagen Remodeling, improved fertility	Xin et al. (2020)
LPS-pre- MSCex	NF-κB NLRP3/procaspase 1 signaling	*In vivo* mice model	Prolonged graft survival	Zhang et al. (2023b)
LPS-pre- MSCex	TLR4/NF-κB/STAT3/AKT regulatory signaling pathway	*In vitro/in vivo* mice model	Alleviation of inflammation and enhanced diabetic cutaneous wound healing	Ti et al. (2015)
Melatonin pretreated MSCex	Upregulating the expression of PTEN and inhibiting the phosphorylation of AKT	*In vitro/in vivo* mice model	Angiogenesis, collagen synthesis, diabetic wound healing	Liu et al. (2020)
MSCex	Regulatory effect of miR-148a through KLAF6/STAT3 signaling	*In vitro/in vivo* mice model	Protection against liver fibrosis	Tian et al. (2022)
MSCex	miRNA-21-5p	*In vitro/in vivo* mice model	Repairing myocardial injury	Shen and He (2021)
MSCex	miRNA-182	*In vitro/in vivo* mice model	Repairing cardiac injury	Zhao et al. (2019)
TNF-α**-**pre**-**gingival tissue-derived MSCex	miRNA-1260b targeted Wnt5a-mediated RANKL pathway	*In vivo* mice model	Preventing bone loss in the periodontal tissue	Nakao et al. (2021)
TNF-α**-**pre**-**MSCex	JAK/STAT pathway regulated by galactin-1	Electric tool scratching IUA mice model	Improved therapeutic effect	Li et al. (2022a)
Human adipose derived MSCex	p38 MAPK/NF-κb	*In vitro/In vivo* rat model	Improved facial nerve injury	Xue et al. (2024)
Human jaw bone marrow derived MSCex	miRNA-223 targeting pknox1	*In vitro/In vivo* mice model	Enhanced wound healing	He et al. (2019b)
IL-1β-pretreated mouse bone marrow derived MSCex	miRNA-21	*In vitro/In vivo* mice model	Mitigated sepsis	Yao et al. (2021)
Mice bone marrow derived MSCex	miRNA-21a-5p targeting KLF6 and ERK1/2 signaling pathways	*In vitro/In vivo* mice model	Attenuated atherosclerosis	Ma et al. (2021)
Rat bone marrow derived MSC^NIC^-exo	miRNA-125a-5p targeted TRAF6/IRF5 signaling pathway	*In vitro/In vivo* mice model	Cardiac repair	Gong et al. (2024)
MSC derived ex	CD74 regulating TSC2-mTOR-AKT pathway	*In vitro/In vivo* mice model	Improved therapeutic strategy for abdominal aortic aneurysm	Xu et al. (2024)
Mice adipose tissue derived MSCex	Let-7c	*In vitro/In vivo* mice model	Promoted survival rate of fat grafts	Hao et al. (2021)
Mice PBMSCex	miRNA-135b regulated MAPK6 pathway	*In vitro/In vivo* mice model	Improved cartilage injury	Wang and Xu (2021)
human MSCexs	tsRNA-21109	*In vitro/In vivo* mice model	Alleviated systemic lupus erythematosus	Dou et al. (2021)
Mice adipose derived MSCexs	miRNA-150–5p targeting Irs1 and down-regulating the e PI3K/Akt/mTOR pathway	*In vitro/In vivo* mice model	Mitigated sepsis	Zheng et al. (2024)
Human hucMSCexs	miRNA-146a-5p via regulating TRAF6 and STAT1 signal pathways	*In vitro/In vivo* mice model	Restored renal function	Zhang et al. (2022c)

#### 2.1.4 Natural killer cells

Natural Killer cells (NK) are the part of the innate immune system but also exhibit traits associated with the adaptive immune system. These include the expansion of pathogen-specific cells, the formation of long-lasting memory cells that can persist following encounters with cognate antigens, and the capacity to elicit an enhanced secondary recall response upon re-challenge (Gianchecchi et al., 2018). MSCex primarily exert an immunosuppressive effect on NK cells, influencing their proliferation, activation, and cytotoxicity. In a rat model of experimental autoimmune uveitis, the administration of MSCex around the eye mitigated EAU-induced damage by reducing the trafficking of CD161+NK cells to the lesion (Bai et al., 2017). Another research indicates that exosomes originating from fetal liver MSCs possess the ability to suppress the proliferation, activation, and cytotoxic effects of NK cells. Exosomes include components such as latency associated peptide (LAP), TGFβ, and thrombospondin 1 (TSP1), which initiate TGFβ signaling in NK cells. Neutralizing TGFβ with an antibody counteracted the inhibitory effects, suggesting that TGFβ serves as a crucial mediator in this process (Fan et al., 2019) (Figure 2).

#### 2.1.5 Dendritic cells

Dendritic cells (DC) represent a category of cells derived from bone marrow, originating from lympho-myeloid hematopoiesis. DCs play a crucial role in coordinating innate inflammatory responses and adaptive immunity by activating T-cells (Morel and Butterfield, 2015). MSCex inhibit antigen uptake and maturation of immature DCs, resulting in reduced expression of activation markers and decreased production of pro-inflammatory cytokines. MSCex also enhance the release of the anti-inflammatory cytokine TGF-β. MSCex-treated DCs demonstrate reduced migration in response to specific signals; however, they maintained the capacity to stimulate T cell proliferation (Reis et al., 2018). Another study demonstrated that exosomes derived from MSCs modulate DCs to enhance an immature phenotype, resulting in increased production of anti-inflammatory cytokines such as IL-10 and IL-6. Co-cultures of conditioned DCs with T cells exhibited diminished levels of IFN-γ and a reduced population of Th17 cells, whereas the quantity of regulatory T cells increased (Favaro et al., 2016). Mehri Shahir and the colleagues investigated the impact of exosomes obtained from adipose derived MSCs on dendritic cells DCs. Treatment of DCs with MSCex resulted in a decrease in surface marker expression, suggesting a suppression of maturation. Exosomes also decreased the release of the pro-inflammatory cytokine IL-6 and increased the levels of the anti-inflammatory cytokines IL-10 and TGF-β. The treatment resulted in reduced lymphocyte proliferation. MSCex significantly influence dendritic cell function and may regulate immune responses (Shahir et al., 2020) (Figure 2).

### 2.2 Modulation of apoptosis and autophagy

#### 2.2.1 MSCex in regulating apoptotic pathways

MSCex inhibit the proliferation, arrest cell cycle progression, promote apoptosis, and inhibit differentiation of myeloid cells (Liu X. et al., 2023). These can safeguard several cell types from apoptosis by transporting anti-apoptotic molecules such as miRNAs and proteins that modulate essential apoptotic pathways (Lai et al., 2010; Gatti et al., 2011). These have shown their therapeutics efficiency in several injuries including, heart injury, cancer, reproductive ailments, bone and joint fractures, and brain and nerve injuries mediating the process of apoptosis. MSCex confer protection to heart cells against apoptosis in low oxygen conditions by delivering miR-144, which targets the PTEN/AKT pathway, thereby enhancing p-AKT levels and decreasing cell death (Wen et al., 2020). Another study showed miR-486-5p suppresses PTEN expression and activates the PI3K/AKT pathway, resulting in decreased apoptosis and protection of myocardial cells from ischemic injury (Sun X. H. et al., 2019). MSCex protect cardiac cells against apoptosis through the delivery of miR-210, which specifically targets AIFM3 (Cheng et al., 2020). Exosomes produced from bone marrow MSCs were observed to enhance the Th1/Th2 ratio and facilitate apoptosis in acute myeloid leukemia (AML) cells. It was demonstrated that miR-222-3p targeted IRF2, amplifying these effects, while IRF2 could partially reverse the impact of miR-222-3p (Yuan et al., 2023). hsa-miR-143-3p in exosomes produced from human mesenchymal stem cells (hMSCs) was found to diminish pancreatic cancer. hsa-miR-143-3p was identified as being upregulated in hMSCex, which inhibited cancer cell growth and invasion while triggering apoptosis *in vitro*. *In vivo*, hsa-miR-143-3p suppressed tumor proliferation in murine models (Wang et al., 2021). Several studies have demonstrated the role of MSCex mediating apoptosis for reproductive therapy. A study assessed the therapeutic efficacy of hMSCexo in enhancing ovarian function in a mouse model of natural ovarian aging. The results indicated that hMSCexo reinstated follicle counts and hormone concentrations while suppressing PTEN expression, hence diminishing apoptosis in aging ovaries. miR-21-5p, a microRNA with elevated expression in exosomes, was recognized as a crucial element in inhibiting PTEN and maintaining ovarian function (Li et al., 2023). The MSCex treatment for erectile dysfunction in a rat model of cavernous nerve injury markedly boosted erectile function, augmented smooth muscle content, and elevated neuronal nitric oxide synthase levels in the corpus cavernosum. The intervention also decreased apoptosis in smooth muscle cells and enhanced the smooth muscle-to-collagen ratio (Ouyang et al., 2018). MSCex regulate the process of apoptosis in skeleton diseases such as osteoarthritis (OA)and intervertebral disc injury. A study examines the role of exosomal lncRNA-KLF3-AS1 derived from hMSCs in enhancing chondrocyte proliferation in OA via the miR-206/GIT1 pathway. MSCex inhibited induction of apoptosis, promoted chondrogenic markers, diminished hypertrophy markers, and safeguarded chondrocytes against IL-1β-induced damage (Liu et al., 2018). Qi and the colleagues demonstrated that BM-MSCex were taken up by chondrocytes *in vitro*, which improved cell viability and reduced apoptosis in inflammatory conditions induced by IL-1β. BM-MSCex lessened mitochondrial damage and altered the phosphorylation of critical signaling pathways, including p38, ERK, and Akt (Qi et al., 2019). The research investigates the role of MSCex in facilitating cartilage repair within osteochondral defects. MSCex attenuated the apoptosis, promoted cell proliferation, infiltration, matrix synthesis, and initiated a regenerative immune response. The observed effects were facilitated by exosomal CD73-mediated activation of adenosine, which in turn activated the AKT and ERK signaling pathways, crucial for enhanced cell migration and proliferation (Zhang S. et al., 2018). Impact of MSCex on endplate chondrocytes in the context of intervertebral disc degeneration was also examined. MSC-exosomes were observed to decline apoptosis and calcification of endplate chondrocytes in conditions of oxidative stress, with these effects mediated by miR-31-5p, which regulates ATF6-related endoplasmic reticulum stress (Xie et al., 2020). MSCex, harboring miR-21, have been shown to inhibit nucleus pulposus cells apoptosis by delivering miR-21 to nucleus pulposus cells, resulting in the suppression of PTEN and the activation of PI3K/Akt pathway. In both cellular and rat models, MSCex markedly decreased NPC apoptosis and attenuated intervertebral disc degeneration (Cheng et al., 2018).

#### 2.2.2 Autophagy induction and its protective effects in inflammatory conditions

**FIGURE 3:**
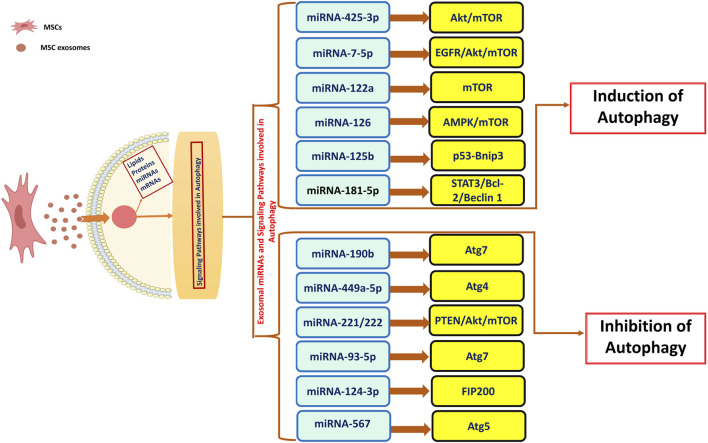
Role of MSCex miRNAs in Autophagy. MSCex enriched with miRNA cargo induces/inhibits the process of autophagy via various signaling pathways.

Autophagy is essential in inflammatory diseases as it regulates cellular homeostasis and immune responses. This process facilitates the elimination of damaged organelles and proteins, thereby decreasing oxidative stress and inflammation. In conditions like IBD, rheumatoid arthritis and neurodegenerative disorders, impaired autophagy facilitates disease progression through the accumulation of damaged cells and the promotion of chronic inflammation (Levine and Kroemer, 2008). MSC transplantation is an effective approach in regenerative medicine for the repair of injured organs through the modulation of autophagy. Prior studies have emphasized the therapeutic efficacy of exosomes obtained from MSCs in reducing the advancement of cisplatin-induced acute nephrotoxicity. Research indicates that exosomes can reduce apoptosis markers and inflammation-related cytokines, mainly via the overexpression of the 14-3-3ζ protein and its interaction with ATG16L (Dutta et al., 2014; Jia et al., 2018). Recent studies indicate that autophagy activation may have therapeutic effects on cerebral ischemia/reperfusion injury, via the PI3K/Akt/mTOR signaling pathway with its mechanism potentially linked to the AMPK-dependent autophagic flux (Yong et al., 2019; Yao et al., 2019; He H. et al., 2019; Zeng et al., 2020). In another study, in both *in vitro* and *in vivo* models, hucMSCex decreased mitochondrial apoptosis and inflammatory cytokines in kidney cells, while enhancing the autophagy marker LC3B and associated genes. The protective effects of hucMSCex were replicated by the autophagy inducer rapamycin and were reversed by an autophagy inhibitor (Wang B. et al., 2017). Exosomes transport diverse cargos and engage with recipient cells through three primary mechanisms including phagocytosis, ligand-receptor binding and membrane fusion. Upon uptake by recipient cells, exosomes release their cargo mainly consisting of lipids, proteins and RNAs, having the potential to modulate autophagy through various signaling pathways regulated by different miRNAs depending upon the exosome cargo (Figure 3).

## 3 Therapeutic applications of MSCex in specific inflammatory diseases

### 3.1 Osteoarthritis

OA is the most prevalent musculoskeletal disorder, resulting in functional impairment. The condition is clinically characterized by joint pain, tenderness, crepitus, stiffness, and limitation of movement, accompanied by infrequent effusion and varying degrees of local inflammation (Felson, 2009). The principal feature of the disease is the degeneration of articular cartilage accompanied by subchondral bone sclerosis. Histologically, the disease is marked by initial fragmentation of the cartilage surface, chondrocyte cloning, vertical clefts within the cartilage, variable crystal deposition, remodeling, and eventual disruption of the tidemark by blood vessels (Madry et al., 2012; Pereira et al., 2015). In progression phase, enzymes like MMP-13 and ADAMTS5 are upregulated, which accelerate matrix degradation and the progression of OA. With the progression of OA, there is an increase in pro-inflammatory mediators such as interleukin-1β (IL-1β) and tumor necrosis factor–α (TNF-α), accompanied by the action of degradative enzymes that inflict damage and inflammation on cartilage tissue. MSCex may function as a viable, cell-free substitute for conventional MSC therapies in cartilage healing. Efficiency of MSCex for cartilage regeneration in a rat model exhibiting osteochondral abnormalities was investigated. Weekly intra-articular exosome injections resulted in substantial regeneration of cartilage and subchondral bone compared to phosphate-buffered saline (PBS) therapy, achieving complete restoration by 12 weeks (Zhang et al., 2016). Bone marrow derived MSC-EVs (BM-MSC-EVs) mitigated inflammation in chondrocytes by suppressing TNF-alpha-induced pro-inflammatory genes and collagenase activity. Furthermore, BMMSC-EVs facilitated cartilage regeneration by augmenting the synthesis of proteoglycans and type II collagen (Vonk et al., 2018).

The established connection between inflammation and the progression of OA suggests that the anti-inflammatory properties of MSCex may provide significant therapeutic advantages for OA management (Xia et al., 2014). Various reviews have been published describing the therapeutic role of MSCex in OA (Malekpour et al., 2022; Mianehsaz et al., 2019; Gerami et al., 2023; Kim et al., 2021; Mohd Noor et al., 2021; Rizzo et al., 2023; Wang et al., 2020a). A recent study indicates that hucMSCex facilitate cartilage regeneration in OA through the reduction of inflammation and enhancement of tissue repair mechanisms. In rat OA models, hucMSCex reduced cytokines (TNF-α, IL-1β, IL-6) and inhibited cartilage-degrading enzymes (MMP-13, ADAMTS-5), while promoting collagen II expression (Yang H. et al., 2024). Another study found that MSCex effectively inhibited NLRP3 inflammasome activation in macrophages, resulting in decreased release of pro-inflammatory factors such as IL-1β and IL-18, thereby alleviating OA (Zhou H. et al., 2022). The findings from a previous study indicated that BM-MSC-EVs diminished inflammation by suppressing TNF-alpha-induced pro-inflammatory factors and collagenase activity in OA chondrocytes. BM-MSC-EVs facilitated cartilage regeneration by enhancing the production of proteoglycan and type II collagen (Vonk et al., 2018). Researchers utilized hMSCex, *in vitro* and *in vivo* models, and gene silencing to demonstrate that exosomal KLF3-AS1 mitigated cartilage damage, suppressed chondrocyte apoptosis, and facilitated cartilage regeneration, indicating as a potential therapeutics for OA (Liu et al., 2018). Exosomes generated from human embryonic stem cell-induced mesenchymal stem cells (ESC-MSCex), administered to an OA mouse model, mitigated cartilage breakdown and preserved chondrocyte viability by augmenting collagen II synthesis and diminishing ADAMTS5 expression in the presence of interleukin 1 beta (IL-1β) (Wang Y. et al., 2017). Another study showed similar findings using exosomes derived from humane bone marrow derived mesenchymal stem cells (BM-MSCex). This research showed that BM-MSCex facilitated cartilage regeneration, enhanced chondrocyte proliferation, and alleviated pain in OA mice. *In vitro*, exosomes mitigated the effects of IL-1β on chondrocytes, whereas *in vivo*, they enhanced cartilage health and alleviated pain responses in OA rats (He et al., 2020). Proteins harbored by the MSCex have also been known to be the potential elements in relieving OA. EVs derived from hucMSCs diminished inflammation, enhanced chondrocyte vitality, and decelerated OA progression in a murine model by facilitating chondrocyte proliferation and migration while suppressing apoptosis. The METTL3 protein was recognized as a crucial element in these protective actions, rendering EVs a possible therapeutic alternative (Zhou H. et al., 2022). Another study examined the effects of BM-MSCex on chondrocyte viability in inflammatory conditions, demonstrating that can inhibit chondrocyte apoptosis and mitochondrial damage caused by IL-1β. BM-MSCex modulated essential pathways, including p38, ERK, and Akt (Qi et al., 2019). The role of MSCex in the repair of temporomandibular joint OA (TMJ-OA) in rats is significant, as they demonstrate the capacity to alleviate pain, reduce inflammation, and mitigate degeneration, while also facilitating the restoration of cartilage and bone. Exosomes promote joint repair by activating adenosine signaling pathways (AKT, ERK, and AMPK), which enhance matrix synthesis and suppress inflammatory factors (Zhang S. et al., 2019). Macrophage polarization is another way followed by the MSCex alleviating the OA. Research indicates that MSCex can attract M2 macrophages to infiltrate joint cavities in OA, while simultaneously decreasing M1 macrophage infiltration, downregulating IL-1β and TNF-α expression, and effectively managing the progression of OA (Zhang S. et al., 2018). Role of RNAs harbored by the MSCex in alleviating OA following various immunomodulatory effects including macrophage polarization is given in Table 2. Exosomes derived from various types of cells have varying therapeutic potential depending on their cargo. RNA sequencing and comparative analysis have identified notable compositional differences in exosomes derived from human adipose tissue and bone marrow MSCs. This includes specific tRNA species, such as Nanog, POU5F1A/B, and Sox2 expression, which seem to correlate with the differentiation status and tissue origin of the MSCs (Börger et al., 2017; Baglio et al., 2015). A research assessed AD-MSCex and BM-MSCex in relation to alleviating symptoms of OA. Exosome-treated OA mouse models exhibited enhanced collagen II expression, with BM-MSCex demonstrating superior outcomes compared to AD-MSCex in both gene expression and tissue analysis (Fazaeli et al., 2021). Recent study compared the therapeutic properties of human MSCs derived from adipose tissue, bone marrow, and endometrium in a myocardial infarction model demonstrated that endometrial MSCs offered enhanced cardio-protection (Wang et al., 2016). The findings indicate that intrinsic differences among exosomes derived from MSCs of different origins must be carefully considered, as they may substantially affect therapeutic outcomes.

**TABLE 2:** Role of miRNA derived from MSCex in alleviating OA and the subsequent mechanisms.

Type of miRNA	Source of exosomes	Model	Function	Ref.
miRNA-92a-3p	BM-MSCs	*In vitro*	Regulates cartilage development and homeostasis by directly targeting WNT5A	Mao et al. (2018)
miRNA-320c	BM-MSCs	*In vitro*	Upregulates SRY-Box 9 Downregulates MMP-13 Enhances chondrocyte proliferation	Sun et al. (2019b)
miRNA-135b	BM-MSCs	*In vitro* and *In vivo*	Promotes M2 polarization of synovial macrophage and improves cartilage damage	Wang and Xu (2021)
miRNA-140- 5p	Synovial-MSCs	*In vitro* and *In vivo*	Enhances the proliferation and migration of chondrocytes	Tao et al. (2017)
miRNA-101	BM-MSCs	*In vitro*	Upregulates the osteogenic differentiation of MSCs	Li et al. (2021)
miRNA-210	BM-MSCs	*In vitro*	Improves the proliferation of chondrocyte and inhibits apoptosis	He et al. (2020)
miRNA- 23b	BM-MSCs	*In vitro*	Induces cartilage differentiation	Ham et al. (2012)
miRNA-100- 5p	Infrapatellar fat pad -MSCs	*In vitro and* *In vivo*	Protects articular cartilage from injury and improves gait abnormality in OA mice	Wu et al. (2019)
miRNA-129- 5p	Synovial-MSCs	*In vitro*	Regulates the inflammatory response and reduces apoptosis of chondrocytes	Qiu et al. (2021)
miRNA-9-5p	BM-MSCs	*In vitro and* *In vivo*	Regulates SDC1 for anti-inflammatory and chondro-protective effects	Cosenza et al. (2017)
miRNA-136- 5p	BM-MSCs	*In vivo*	Enhances the chondrocyte proliferation and inhibits chondrocyte degeneration	Chen et al. (2020)
miRNA-146b-5p	BM-MSCs	*In vitro and* *In vivo*	Inhibits activation of TRAF6, suppresses the inflammatory responses and extracellular matrix degradation, promotes chondrocyte autophagy for the protection of osteoarthritic cartilage cells	Lou et al. (2023)
miRNA-3473b	BM-MSCs	*In vitro and* *In vivo*	Promotes migration, improves anabolism and inhibits apoptosis of chondrocytes	Zhang et al. (2023c)
microRNA-29a	BM-MSCs	*In vivo*	Exerts anti-inflammatory effects and maintains extracellular matrix stability	Yang et al. (2024b)
miRNA-181c-5p	hucMSCs	*In vitro and* *In vivo*	Induces proliferation, migration and differentiation potentials of BMSCs	Zhang et al. (2022d)
miR-125a-5p	BM-MSCs	*In vitro and* *In vivo*	Regulates the chondrocyte migration and inhibits degeneration	Xia et al. (2021)

### 3.2 Inflammatory bowel disease

IBD is mostly categorized into ulcerative colitis (UC) and Crohn’s disease (CD) (Zhang and Li, 2014). UC is defined by inflammation restricted to the colon, commencing in the rectum and progressing continually in a proximal manner and frequently involving the peri-appendiceal region (Baumgart and Sandborn, 2007). Conversely, CD can impact any segment of the gastrointestinal tract; most frequently the terminal ileum or perianal area in a sporadic and discontinuous fashion (Guan, 2019). CD is often linked to complications such as strictures, abscesses, and fistulas, in contrast to ulcerative colitis (Khor et al., 2011). The pathogenesis of IBD entails a complex interaction of genetic, environmental, microbial, and immunological factors. Genetic predisposition, characterized by more than 160 known susceptibility loci, including prominent genes such as NOD2, ATG16L1, and IL23R, significantly influences disease susceptibility by altering immune responses and interactions with gut microbiota (Liu et al., 2015; Graham and Xavier, 2021). Environmental variables, including smoking, food, antibiotics, stress, and vitamin D deficiency, significantly impact the start and progression of disease by altering immune function and gut barrier integrity (Singh and Bernstein, 2022). Microbial imbalances, leading to the diminished biodiversity in the gut microbiome and the presence of pathogenic strains, disturb intestinal homeostasis, hence fostering inflammation (Zubair et al., 2024). Immune dysregulation is pivotal in the pathophysiology of IBD, characterized by specific Th1/Th17 immune responses in CD and atypical Th2 responses in UC, driven by cytokines such as IFN-γ, IL-13, and IL-17 (Geremia et al., 2014; Jiang et al., 2022). Furthermore, impairments in the mucosal barrier and autophagy processes increase intestinal permeability, promoting chronic inflammation (Lee and Eun, 2022).

The anti-inflammatory and immunomodulatory properties of MSCex support the development of innovative cell-free treatments for IBD. Increasing evidence indicates that MSCex may serve as a potential candidate for the treatment of IBD (Nazari et al., 2022; Yu et al., 2021; Wu et al., 2018; Mao et al., 2017). Administration of MSC-EVs, whether local or systemic, in dextran sodium sulfate (DSS)-induced mouse models has been shown to enhance clinical outcomes in colitis, resulting in improved survival rates and reduced gut inflammation as evidenced by histopathological analyses (Munoz-Perez et al., 2021). MSCex serve as a multi-functional treatment approach, offering anti-inflammatory, regenerative, and anti-fibrotic effects for the effective management of IBD. MSCex specifically target immune cells, comprising macrophages, T lymphocytes, and dendritic cells, which are crucial in the inflammatory processes associated with IBD. Administering the MSCex in DSS induced acute colitis model, the concentrations of IFN‐γ, TNF‐α, IL‐12, and IL‐17 were decreased, whereas the levels of TGF‐β, IL‐4, and IL‐10 were elevated in the lymph nodes and spleen of mice administered exosomes. The proportions of CD4^+^ CD25^+^ Foxp3+ Treg cells increased in the lymph nodes and spleens of mice (Heidari et al., 2021).

**FIGURE 4:**
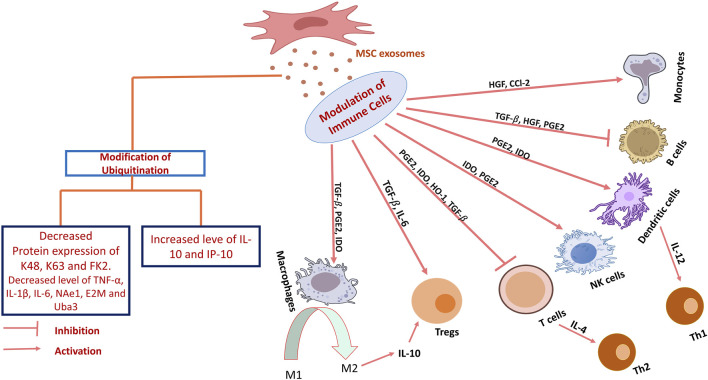
Anti-inflammatory effect of MSCex in treating IBD. MSCex exert their therapeutic response directly through immuno-modulation or ubiquitination pathways.

MSCex facilitate the transition of macrophages from a pro-inflammatory (M1) phenotype to an anti-inflammatory (M2) phenotype, thereby diminishing inflammation (Arabpour et al., 2021). They also influence T cells by inhibiting pro-inflammatory Th1 and Th17 cells and promoting Tregs, thereby aiding in the balance of the immune response (Chen et al., 2016). Figure 4 illustrates the anti-inflammatory pathways including the process of ubiquitination followed by MSCex in relieving IBD. MSCex are also known to alleviate the harmful bacteria playing their vital role in IBD progression (Qiao et al., 2024). The secretome of dental pulp multipotent MSCs has been shown to inhibit the invasion of *Fusobacteria* in the oral cavity (Ravenscroft et al., 2022). The infusion of both hucMSCex and human fetal placenta exosomes has been shown to decrease the prevalence of pro-inflammatory intestinal bacteria, including *Verrucomicrobia* and *Akkermansia muciniphila*, thereby improving colitis (Yan et al., 2023). MSCex contains various proteins that suppress colitis activity, particularly metallothionein-2 (MT-2), which mitigates the intestinal inflammatory response by preserving the integrity of the intestinal barrier and promoting M2b macrophage polarization (Liu H. et al., 2019). Another study revealed the TSG-6 protein harbored by the hucMSCex as potential therapeutic target for IBD. It improved the epithelial integrity, enhanced anti-inflammatory response and altered the activity of Th2 and Th17 cells in the mesenteric lymph nodes in a DSS and 2,4,6-trinitrobenzenesulfonic acid (TNBS) induced mouse models (Yang et al., 2021). However, exosomal miRNAs are known to be more promising as future therapeutics approach. It was found that differentially expressed exosomes from MSCs reduce pyroptosis and improve UC, with optimal results at 400 μg per mouse twice weekly. Exosomal miRNAs appear to inhibit pyroptosis via tumor necrosis factor-related apoptosis inducing ligand signaling and IFN-gamma pathways (Chang et al., 2022). A study showcased that hucMSCex are enriched with miR-378a-5p and can reduce colitis by inhibiting macrophage pyroptosis associated with NLRP3 inflammasome activation. In a DSS-induced colitis model and *in vitro* experiments, hucMSCex decreased inflammation by inhibiting IL-18, IL-1β, and caspase-1, thereby improving cell survival (Cai et al., 2021). Another research demonstrated that BM-MSCex, particularly those primed with IFN-γ, are abundant in miR-125a and miR-125b and exert therapeutic effects on colitis by inhibiting Th17 differentiation and enhancing Treg cells through targeting Stat-3 (Yang et al., 2020). Li et al. revealed that exosomal miR-181a derived from MSCs has the potential to mitigate experimental colitis through the enhancement of intestinal barrier function. It demonstrated anti-inflammatory properties and altered the gut microbiota (Gu et al., 2021). It has been shown that 3D culture of MSCex instead of traditional 2D culture is more promising for IBD therapy. In this context, a study demonstrated exosomes derived from 3D culture of MSCs improved anti-inflammatory effects in a ligature-induced periodontitis model by re-establishing the balance between reactive Th17 cells and Tregs in inflamed periodontal tissues through the miR-1246/Nfat5 axis (Zhang Y. et al., 2021).

### 3.3 Respiratory diseases

MSCex hold potential for the treatment of various pulmonary disorders, including asthma, idiopathic pulmonary fibrosis (IPF), pulmonary artery hypertension and acute respiratory distress syndrome (ARDS) (Abreu et al., 2016; Cruz et al., 2015). Exosomes possess advantages like diminutive size (approximately 100 nm) for aerosol inhalation in treating airborne infections. MSCex limit pro-inflammatory pathways, contributing to the remodeling of inflammatory lung disease and decreasing oxidative stress and pulmonary fibrosis (Fujita et al., 2018). MSCex, especially their microRNA content, are under investigation for the treatment of respiratory diseases, with research concentrating on pulmonary delivery methods such as inhalation and intra-tracheal instillation (Azhdari et al., 2022). Ad-MSCex effectively transfer the mitochondrial components to the pulmonary microenvironment, enhancing macrophage mitochondrial integrity and oxidative phosphorylation levels. This process facilitates the restoration of metabolic and immune homeostasis in airway macrophages, thereby alleviating lung inflammatory pathology (Xia et al., 2022).

#### 3.3.1 Asthma

Asthma is a prevalent condition characterized by chronic inflammation of the lower respiratory tract. Chronic lower airway inflammation is more prevalent in individuals with inflammatory disorders of the upper airway (Mims, 2015). Asthma is a chronic respiratory condition impacting over 300 million individuals globally, with its prevalence rising annually (Backman et al., 2017). Airway inflammation and remodeling are fundamental components of asthma, leading to histological alterations in airway structure. These alterations include thickening of the airway basement membranes, proliferation of smooth muscle, and increased fibrosis, ultimately resulting in a decline in lung function (Lambrecht and Hammad, 2015). MSCs exhibit potential for treating lung injuries, attributed to their immunomodulatory and regenerative properties, primarily through the secretion of EVs. These EVs exhibit reduced immunogenicity and tumor risk, presenting benefits compared to stem cells (Abbaszadeh et al., 2020). A study examined the therapeutic effects and mechanisms of MSCex in severe steroid resistant asthma (SSRA), revealing that intra-tracheal administration of MSCex diminished airway hyper-responsiveness, inflammation, and tissue alterations in SSRA mice. MSCex influenced macrophage polarization, favoring anti-inflammatory M2 over pro-inflammatory M1 polarization, with TRAF1 recognized as a crucial regulatory protein (Dong B. et al., 2021). Another research examined the impact of EVs derived from hypoxia-conditioned MSCs on asthma. In a murine asthma model, Hypo-EVs demonstrated greater efficacy than normoxic EVs (Nor-EVs) in mitigating airway inflammation, remodeling, and fibrosis. EVs derived from hypoxia-conditioned MSCs reduced pro-fibrogenic markers (α-SMA, collagen-1, and TGF-β1-p-smad2/3 signaling) while increasing miR-146a-5p, which is critical for their protective effects (Dong L. et al., 2021). Intranasal of administration of MSCex in asthmatic mice revealed that MSCex may protect against allergic asthma by increasing IL-10-producing interstitial macrophages, likely originating from the spleen (Ren et al., 2021). In addition, MSCex increase the levels of IL-10 and TGF-β1 in peripheral blood mononuclear cells (PBMCs) derived from asthmatic patients, thereby promoting Treg proliferation and immune-suppressive activity mediated by antigen-presenting cells (Du et al., 2018). Recently, Jung et al. evaluated the impact of adipose stem cell (ASC)-derived EVs on cytokine concentrations and Tregs in PBMCs of individuals with asthma. ASC-derived EVs diminished IL-4 and co-stimulatory molecules (CD83 and CD86) in PBMCs, concurrently elevating TGF-β and Treg expression indicating immunomodulatory effects of ASC-derived EVs, facilitating Treg expansion and diminishing inflammation in asthmatic patients (Jung et al., 2024). MiRNAs harbored by the MSCex have shown wide impact in asthma reduction (Liu et al., 2024). A study showed that miR-1470 in MSCex has been shown to promote the differentiation of CD4^+^CD25+FOXP3+ Tregs in asthmatic patients by upregulating p27kip1, a regulator of cyclin-dependent kinases that governs cell differentiation programs (Zhuansun et al., 2019). Furthermore, MSCex miR-146a-5p has been shown to impair the function of ILC2s, decrease inflammatory cell infiltration, reduce pulmonary mucus production, and lowering Th2 cytokine secretion, thereby mitigating airway hyper-responsiveness in a mouse model characterized by Group 2 innate lymphoid cells (ILC2) dominance in asthma (Fang et al., 2020). miR-188 from MSCex inhibits JARID2/Wnt/β-catenin axis, thus inhibiting mucus production, inflammatory cell infiltration, and collagen deposition in the lung tissues of asthmatic mice (Shan et al., 2022). Exosomal miR-301a-3p derived from air way smooth muscle cells (AMSCs) reduces the secretion of inflammatory factors such as TNF-α, IL-1β, and IL-6 in ASMCs exposed to platelet-derived growth factor. This effect is accompanied by inhibited proliferation and migration of AMSCs, leading to a reduction in airway inflammation and remodeling in an OVA-induced asthma mouse model (Feng et al., 2022).

#### 3.3.2 Acute respiratory distress syndrome (ARDS)

ARDS is a severe respiratory condition marked by bilateral chest radiographic opacities and significant hypoxaemia resulting from non-cardiogenic pulmonary edema manifested by swiftly advancing dyspnea, tachypnea, and hypoxemia (Meyer et al., 2021; Saguil and Fargo, 2020).The pathogenesis of ARDS is intricate and entails the activation and deregulation of numerous interconnected pathways of injury, inflammation, and coagulation, occurring both in the lungs and systemically (Bos and Ware, 2022). MSCs and derived exosomes have been explored for their influence as therapeutic target in ARDS. Patients with ARDS exhibited a greater presence of MSC-EVs compared to control subjects. The EVs included the Runx1 transcription factor, with a higher ratio of Runx1p66 to Runx1p52 correlating with improved survival outcomes. Runx1p66 has been demonstrated to facilitate endothelial cell proliferation, improve junctional integrity, and mitigate lung injury in LPS-treated models, indicating a protective function in the recovery from ARDS (Shah et al., 2018). Moreover, in ARDS environment, MSCs facilitate an anti-inflammatory and phagocytic macrophage phenotype via EV-mediated mitochondrial transfer. The alterations in macrophage phenotype induced by MSCs are fundamentally dependent on the enhancement of oxidative phosphorylation in macrophages. Alveolar macrophages administered MSC-EVs demonstrate improved outcomes in lung injury models *in vivo* (Morrison et al., 2017). MSCex may function as an innovative treatment for COVID-19-induced ARDS by utilizing their immunomodulatory and tissue healing capabilities. Exosomes may mitigate the cytokine storm linked to COVID-19, diminish pulmonary inflammation, and facilitate tissue healing, circumventing the complications of direct cell therapy (Taghavi-farahabadi et al., 2020). It was observed that the treatment of hucMSC-MVs enhanced survival and reduced bacterial proliferation, pulmonary inflammation and protein permeability in *Escherichia coli*-induced pneumonia experimental model (Monsel et al., 2015). Recently, *ex vivo* model of severe bacterial pneumonia utilizing perfused human lung tissue, the intra-venous administration of MSC-EVs reduced bacterial load, protein permeability and lung edema, while restoring alveolar fluid clearance (Park et al., 2019). In LPS-induced ARDS animal models, intra-tracheal administration of hucMSC-MVs diminished pulmonary edema (Zhu et al., 2017; Wang et al., 2020b). Another study indicated that MSC-EVs enhanced alveolar-capillary barrier integrity in ARDS by reinstating mitochondrial activity, primarily via mitochondrial transfer. MSC-EVs mitigated lung injury and restored mitochondrial respiration in both cellular and animal models, underscoring their potential as a cell-free therapeutic approach for maintaining lung barrier integrity in ARDS (Silva et al., 2021). Engineered MSCs derived small extracellular vesicles (sEVs) secrete neurotrophic and immunomodulatory factors (MSCex-NTF) and were used in LPS induced ARDS mouse model. MSCex-NTF markedly diminished lung injury, neutrophil infiltration, and production of pro-inflammatory cytokines including IFN-γ, IL-6 and TNF-α in broncho-alveolar lavage fluid, concurrently enhancing blood oxygenation. The findings indicate that MSCex-NTF may serve as a viable therapeutic option for ARDS (Kaspi et al., 2021). In a mice model with *E. coli* endotoxin-induced acute lung injury (ALI), MSC-MVs significantly decreased lung protein permeability, pulmonary edema, neutrophil influx, and markers of inflammation. The therapeutic effects were partially contingent upon KGF Mrna (Zhu et al., 2014). Exosomal miRNAs playing role in ARDS therapy have been studied extensively (Azhdari et al., 2022). Study revealed that MSC-EVs substantially influence human macrophages to diminish inflammation in ARDS through a SOCS1-dependent mechanism. EVs containing miR-181a diminish PTEN expression, hence activating pSTAT5 and enhancing SOCS1 levels in macrophages, both *in vitro* and *in vivo* (Su et al., 2021). Another research explored that hucMSCex derived miR-451 effectively reduced TNF-α, IL-1β, and IL-6 production and improved ALI via TLR4/NF-κB signaling pathway (Liu J. S. et al., 2019). Moreover, MSC-EVs alleviate ALI by transferring miR-27a-3p to alveolar macrophages. Exosomal miR-27a-3p targets NFKB1 and serves as a critical regulator of M2 macrophage polarization (Wang et al., 2020b). Shen et al. found that miR-125b-5p in AD-MSCex may mitigate inflammation-induced ferroptosis in pulmonary microvascular endothelial cells during sepsis-related ALI by modulating Keap1/Nrf2/GPX4 expression (Shen et al., 2023). miR-146a-5p from hucMSCex exerts anti-inflammatory effect in sulfur mustard-induced acute lung injury through targeting targeted TRAF6 (Pei et al., 2023). Role of miRNAs in treating ARDS have been previously reviewed discussing the mechanisms involved in reduction of ALI (Azhdari et al., 2022; Su et al., 2021; dos Santos et al., 2024).

### 3.4 Neurological inflammatory diseases

**FIGURE 5:**
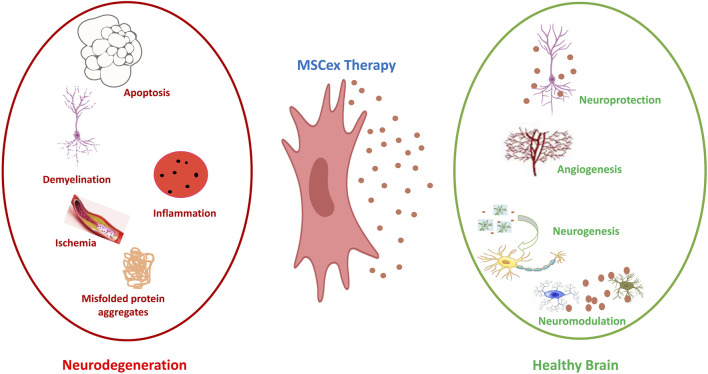
Neuroprotective effect of MSCex. On the left side (Red), the figure illustrates the key pathological mechanisms associated with inflammatory neurodegenerative diseases, including apoptosis, demyelination, ischemia, inflammation and aggregates of misfolded proteins. Middle part (Blue) illustrates the MSCex as therapeutic tools for inflammatory neurodegenerative diseases. The right side of the figure (Green) illustrates the effect of MSCex in protecting and restoring the brain health via the processes of neuroprotection, angiogenesis, neurogenesis and neuro-modulation.

Neurodegenerative diseases constitute an increasing challenge for healthcare systems globally. MSCs exhibit potential therapeutic benefits attributed to their neuro-regenerative, neuroprotective, and immunomodulatory properties, which are associated with the bioactive substances they secrete in the form of exosomes or MVs (Giovannelli et al., 2023). MSCex enhance treatment efficacy through the inhibition of pathological processes and the promotion of regeneration (Riazifar et al., 2019). Demyelination in multiple sclerosis is prevented, and apoptosis in stroke, traumatic brain injury, and spinal cord injury is inhibited (Huang et al., 2017; Li Z. et al., 2019; Ni et al., 2019; Otero-Ortega et al., 2019; Zhang et al., 2022a). They also modulate the immune response by reducing pro-inflammatory cytokines and promoting anti-inflammatory factors. Figure 5 represents the potential of MSCex as promising therapeutic tools for neurological inflammatory diseases by mitigating neurodegenerative damage and promoting brain repair. Underlying mechanisms of regeneration mainly involve: neuroprotection, neurogenesis, neuro-modulation, and angiogenesis.

MSCex and EVs playing neuroprotective role in some specific neurological inflammatory diseases is showed in Table 3.

**TABLE 3:** Neuroprotective role of MSCexs in specific inflammatory neurological diseases.

Disease	Source of MSCexs	Mechanism	Ref.
Ischemic stroke	MSCs	miR-21a-5p induces microglial M2 polarization by targeting STAT3	Xin et al. (2022)
Ischemic stroke	BM-MSCs	miR-150-5p reduces neuronal apoptosis, reduces inflammation through TLR5 pathway	Li et al. (2022b)
Ischemic stroke	BM-MSCs	Improves angiogenesis and neurogenesis, and reduces the expression of IL-1β	Xu et al. (2020)
Ischemic stroke	BM-MSCs	Reduces brain infarction and improves neuron survival via IL-33 siRNA/ST2 siRNA	Liu et al. (2023b)
Ischemic stroke	AD-MSCs	Promotes M2 macrophage polarization via inflammatory mediators	Jiang et al. (2018)
Ischemic stroke	hucMSCs	miRNA-146a-5p reduces microglial-mediated neuro-inflammatory response through IRAK1/TRAF6 pathway	Zhang et al. (2021b)
Ischemia-reperfusion injury	BM-MSCs	miR-124-3p attenuates nerve injury induced by SCIRI via Ern1 and M2 macrophage polarization	Li et al. (2020)
Ischemia-reperfusion injury	BM-MSCs	KLF3-AS1 promoted the Sirt1 deubiquitinating to ameliorate cerebral ischemia/reperfusion inflammatory injury via KLF3-AS1/miR-206/USP22 network	Xie et al. (2023)
Cerebral ischemia	BM-MSCs	Exosomes overexpressed with Steroid receptor coactivator-3 (MSCexs-src3) reduce the pro-inflammatory cytokines and improve the neurological performance by inhibiting ferroptosis	Deng et al. (2024)
Multiple sclerosis	hucMSCs	Reduce the proliferation of conventional T cells producing IFN-γ and IL-17 and increases IL-10	Baharlooi et al. (2022)
Multiple sclerosis	hucMSCs	Suppresses the proliferation of peripheral mononuclear blood cells	Baharlooi et al. (2021)
Amyotrophic lateral sclerosis	MSCs	Promote neuron growth, reduce inflammation, and activate antioxidant pathways with miRNAs	Gschwendtberger et al. (2023)
Amyotrophic lateral sclerosis	AD-MSCs	Protect NSC-34 cells from oxidative damage and increasing cell viability	Bonafede et al. (2016)
Amyotrophic lateral sclerosis	AD-MSCs	Downregulate pro-apoptotic proteins (Bax, cleaved caspase-3) and upregulated the anti-apoptotic protein Bcl-2ɑ	Bonafede et al. (2019)

### 3.5 Cardiovascular inflammatory diseases

MSCex serve as a valuable reference for cardiac function repair and clinical applications in cardiac and vascular diseases by modulating cardiomyocytes viability, inflammatory responses, angiogenesis, and ventricular remodeling followed by heart injury (Zhang H. et al., 2023; Pan et al., 2023). In addition, MSCex exhibit beneficial effects akin to cell therapy, particularly in regenerative and neo-vascular processes (Ahmed and Al-Massri, 2022). MSCex diminished the neurological severity and have shown neuroprotective effect in traumatic brain injury and hemorrhagic shock in swine model (Williams et al., 2019). MSCex demonstrate potential in the treatment of inflammatory cardiomyopathy via improving heart’s inflammatory milieu and the modulation of macrophage activity via the JAK2-STAT6 signaling pathway. In a dilated cardiomyopathy mouse model, MSCex demonstrated a reduction in inflammation, enhancement of cardiac function, and a decrease in cell apoptosis (Sun et al., 2018). Furthermore, hucMSCex demonstrate therapeutic potential in coxsackievirus B3-induced myocarditis by mitigating heart injury, inflammation, and apoptosis. Cardiac function is enhanced through the activation of the AMPK/mTOR via upregulating the autophagy proteins LC3II/I, BECLIN-1 and anti-apoptosis protein BCL-2. Degradation of autophagy flux protein P62 and downregulation of apoptosis protein BAX were also the part of regulatory process (Gu et al., 2020). It was demonstrated that exosome treatment during the post-myocardial infarction period enhanced myocardial strength and alleviated adverse ventricular remodeling by diminishing oxidative stress and initiating the PI3K/Akt pathway (Arslan et al., 2013). Moreover, a study demonstrated that the administration of MSCex significantly reduced infarct size in mice. Intramyocardial injection of cardiac stem cells-derived exosomes in mice subjected to ischaemia-reperfusion injury resulted in a 53% decrease in cardiomyocyte-related apoptosis (Chen et al., 2013). As reviewed by Moghaddam and co, exosomal miRNAs have shown extraordinary role in treating myocardial inflammatory diseases. Recent studies indicate that miRNAs derived from stem cells can be transferred through exosomes from transplanted stem cells to recipient cardiac cells, where they regulate multiple cellular processes, including proliferation, apoptosis, stress responses, differentiation, and angiogenesis (Moghaddam et al., 2019). In a murine myocardial infarction (MI) model, BM-MSCex under hypoxic conditionsenhanced cardiac function and diminished the infarction area, primarily attributable to the increased presence of miR-125b-5p. The knockdown of miR-125b in Hypo-Exo resulted in increased infarct size and cardiomyocyte apoptosis due to the inadequate suppression of pro-apoptotic genes p53 and BAK1 (Zhu et al., 2018). Conferring protection against doxorubicin (DOX)-induced cardiomyopathy by enhancing the expression of anti-apoptotic proteins and increasing the survival rate via Akt-Sp1/p53 signaling pathway. Critical miRNAs in MSC-sEVs (miR-199a-3p, miR-424-5p, and miR-21-5p) facilitate this process, enhancing cardiac function and decreasing cell death (Lee et al., 2021). MSCex inhibited atherosclerosis in ApoE−/− mice by preventing macrophage infiltration and promoting M2 macrophage polarization via miRNA-let7 within atherosclerotic plaques (Li J. et al., 2019). A similar study indicated that MSCex containing miR-21a-5p facilitated macrophage polarization and decreased macrophage infiltration by targeting the KLF6 and ERK1/2 signaling pathways (Ma et al., 2021). Another study demonstrated that MSCex reduce myocardial ischemia/reperfusion (I/R) injury in mice by transporting miR-182 and mediating macrophage polarization (Zhao et al., 2019). MSCex containing miRNA-181a are utilized to target myocardial I/R injury. miRNA-181a in MSCex significantly enhanced anti-inflammatory effects and improved myocardial recovery in a mouse I/R model by suppressing immune-related genes (Zilun et al., 2019). A recent study revealed that MSCex containing miR-21-5p safeguards myocardial infarction via targeting YAP1 signaling pathway (Ji and Wang, 2024).

### 3.6 Inflammatory eye diseases

MSCex have emerged as promising therapeutic tools for treating inflammatory eye diseases. Recent research has highlighted their potential in treating the conditions such as uveitis, dry eye disease and retinopathy. Recently it was revealed that BMSCex modulate Treg/Th17 balance and improve corneal integrity to alleviate dry eye disease in mice model. This was achieved via delivering miRNA-21-5p and inhibiting TLR4, hence suppressing MyD88/NF-κB pathway and reducing the inflammation (Zhao et al., 2024). Another research demonstrated that MSCex significantly reduced the inflammation in a rat model of autoimmune uveitis by downregulating Th1 and Th17 responses while promoting the Treg activity (Bai et al., 2017). MSC-EVs derived from corneal stromal stem cells blocked the neutrophil infiltration and promoted corneal regeneration through delivering miRNA to the target cells. MSC-EVs containing miRNA reduced the fibrosis via blocking fibrotic genes Col3a1 and Acta2 and promoting healing process (Shojaati et al., 2019). miRNA-20 harbored by MSCex relieves graft versus host disease-associated dry eye by reducing the inflammation and improving the epithelial recovery in mice and humans through IL-6/IL-6R/Stat3 pathway mediated M1 macrophages to immuno-suppressive M2 macrophage shift (Zhou T. et al., 2022). MSC-EVs administered in mouse model of retinitis pigmentosa protected the photoreceptors and improved the vision. miRNA-146a harbored by MSC-EVs suppressed macrophage activation, inhibited the NF-κB pathway and regulated inflammatory cytokines via targeting Nr4a3 gene (Zhang et al., 2022b). MSCex containing miRNA-222 improved the retinal regeneration in diabetes mellitus mouse model. After treatment, cellular components of the retina were organized into well-defined layers similar to the normal retina (Safwat et al., 2018). Several other studies have shown the importance of MSCex in relieving inflammatory eye diseases (Yu et al., 2016; Luodan et al., 2024; Guo et al., 2023; Zhang W. et al., 2019).

## 4 Challenges in MSCex-based therapeutics

MSCex play significant role in cell free therapy of several diseases and are successfully implemented in regenerative medicine. Although, some challenges need to be discussed before implementing them to cure for some diseases. Excessive use of MSCex in therapeutics demands more production. Increasing production while preserving MSCex quality and functionality is challenging. Several methods are being applied in the industry to enhance the production of MSCex ensuring the quality control. Preconditioning of MSCex increases the MSCex production ensuring enhanced therapeutic potential (Long and Wang, 2024). This method is carried out via various ways including hypoxia (Berniakovich and Giorgio, 2013), drugs/chemical agents (Yu et al., 2020), inflammatory cytokines (IFN-γ, TNF-α) (Song et al., 2017), and genetic modification (Shang et al., 2020). Conventional 2D cultures have limited yields, necessitating improved techniques such as 3D culturing in bioreactors (Yan and Wu, 2020; Ravi et al., 2015; Lee and Lee, 2022). 3D culture system is being widely used for enhanced production of MSCex and improved bioactivity (Yuan et al., 2022). Bioreactors especially, hollow fiber and vertical wheel bioreactors (Jeske et al., 2023) are being applied in the industry for more MSCex production with preserved qualities and functions. Bioreactors are able to produce large quantity of MSCex but loss of the cargo is the disadvantage. Preconditioning with ethanol and using 3D scaffold bioreactors have been shown to enhance the production along with improved efficacy (Patel et al., 2019). 3D-printed scaffold-perfusion bioreactor system increases the production to almost 40–80-fold with improved efficacy witnessed by efficient wound healing and increased CD31^+^ staining in wound bed tissue (Kronstadt et al., 2023). Standardizing the isolation methods to ensure the maintenance of the quality and potency is another challenge (Witwer et al., 2013). Storage of MSCex at – 80ᵒC and lyophilization are considered as best and the most practiced protocols. However, storing at – 80ᵒC and frequent freeze-thaw cycles affect the quality and bioactivity of exosomes (Park et al., 2018; Wu et al., 2021). Minimizing freeze-thaw cycles by small aliquoting may help to reduce such problems. Lyophilization is considered to be more secure method for long term storage avoiding the disadvantages like frequent freeze-thaw (Trenkenschuh et al., 2022; Charoenviriyakul et al., 2018). However, the drawbacks include aggregation and loss of morphology. Such difficulties can be avoided by using cryoprotectants/stabilizers like trehalose (Bosch et al., 2016), sucrose and using hydrogels (Ahmadian et al., 2024). Differentiation of MSC-EVs derived from other sources is difficult and challenges the purity of the isolated MSC-EVs. Application of MSCex for targeted delivery is still challenging (Fuloria et al., 2021). However, several researches have been done involving labeling techniques and MSCex surface modifications to achieve the accuracy but still it needs to be further investigated (Rao et al., 2007). Moreover, the heterogeneity in size and the contents of MSC-EVs makes it difficult to ensure the accuracy in the targeted therapy (Kosanović et al., 2023). Optimal dosing and the preferred routes of administration in humans is challenging and still need to be investigated and verified as different targeted areas require different doses and routes of administration (Forsberg et al., 2020). MSCex demonstrate potential in preclinical models; however, their mechanisms of action remain inadequately understood, particularly in certain therapeutic domains (Elahi et al., 2020; Shimizu et al., 2019). More work can elucidate the systematic role of exosomes in mediating effects such as immuno-modulation and tissue repair. Further investigations are necessary to completely understand and optimize the production, storage and administration of MSCex. This will make the MSCex, a more reliable therapeutic tool in regenerative medicine in future.

## 5 Conclusion

MSCex have shown promising potential as therapeutic targets for their ability to encapsulate the majority of the therapeutic effects of the MSCs themselves. The therapeutic effects occur via the transfer of bioactive proteins, mRNAs, and miRNAs, modifying cellular behaviors and micro-environmental factors especially immuno-modulation leading to the anti-inflammatory response. Exosomes represent a cell-free therapeutic approach, thereby reducing safety concerns associated with the administration of live cells hence, regarded as plausible therapeutic targets for major specific inflammatory diseases, including those affecting cartilage, heart, lungs, brain, and gastrointestinal tract.
